# Action and Object Word Writing in a Case of Bilingual Aphasia

**DOI:** 10.3233/BEN-2012-119006

**Published:** 2012-03-19

**Authors:** Maria Kambanaros, Lambros Messinis, Emmanouil Anyfantis

**Affiliations:** ^1^Cyprus Acquisition TeamDepartment of English StudiesUniversity of CyprusNicosiaCyprus; ^2^Department of NeurologyNeuropsychology SectionUniversity of Patras Medical SchoolPatrasGreece; ^3^Department of Speech and Language TherapyTechnological Educational Institute PatrasPatrasGreece

**Keywords:** Lexical access, dysgraphia, Orthographic Autonomy Hypothesis (OAH), Greek

## Abstract

We report the spoken and written naming of a bilingual speaker with aphasia in two languages that differ in morphological complexity, orthographic transparency and script Greek and English. AA presented with difficulties in spoken picture naming together with preserved written picture naming for action words in Greek. In English, AA showed similar performance across both tasks for action and object words, i.e. difficulties retrieving action and object names for both spoken and written naming. Our findings support the hypothesis that cognitive processes used for spoken and written naming are independent components of the language system and can be selectively impaired after brain injury. In the case of bilingual speakers, such processes impact on both languages. We conclude grammatical category is an organizing principle in bilingual dysgraphia.

## 1. Introduction

Verbs and nouns are universal categories across languages. Verbs express states and events, that is, what happens to things, including actions, whereas nouns refer to entities such as people, animals, objects, and concepts. In Indo-European languages, verbs are usually marked for tense, aspect, mood, and number, while nouns are marked for case, gender, number, and definiteness (among other features). On the basis of their unique core linguistic function, the term verb can be used interchangeably with *action* (*name*) and correspondingly the term noun with *object* (*name*).

Studies of patients with brain damage report differences in processing actions and objects across languages [4]. Dissociations between action and object naming have also been reported in bilingual brain-impaired individuals in both languages spoken [11,12, 21,24]. Such findings can be taken as evidence for the claim that words are organized in the language system according to semantic and grammatical categories, and (perhaps more controversially) that verbs and nouns are linked to specific cortical regions [2,28]. Brain damage can selectively impair verb and noun processing at many levels in the language system: the conceptual level, the semantic-syntactic level, including lemma retrieval, the phonological level, including lexeme retrieval, and the level of articulation (see [22] and references within). The question of whether patterns of dissociation emerge across modalities of processing in bilingual speakers is our focus in the present study.

A small group of studies has reported an effect of word class on written picture naming of actions and objects in individuals with aphasia. Caramazza and colleagues [14–19,33,34] investigated written lexical access for action and object names in monolingual speakers with aphasia and compared their performance to spoken word production. In these cases, the writing performance of patients was typically spared relative to spoken word production [34]. However, a selective deficit for actions can be restricted to writing in some subjects and to speech in others [3] or a selective deficit for objects in speech only [17]. Most intriguingly, the

ISSN 0953-4180/12/$27.50 © 2012 – IOS Press and the authors. All rights reserved same patient may have difficulty with verbs in spoken but not written word production and with nouns in written but not spoken word production [34]. Such results suggest there are modality-specific (spoken or written) input and output components available for writing verbs and nouns in the brain [17].

Few studies have investigated the written production of verbs and nouns (i.e. actions and objects) in bilingual speakers with aphasia. Hernández et al. [12] described spoken and written output of a bilingual speaker with word-finding difficulties due to degenerative neuropathology. Before the onset of the illness, the patient (JPG) was highly proficient in Catalan and Spanish. When he was presented with the same set of action and object pictures, selected to elicit noun and verb production during spoken and written output, he performed better on object than action words in both modalities. A dissociation between action and object naming in written and spoken word output supports the hypothesis that the grammatical category of verbs and nouns is shared for cognitive and neural mechanisms across languages in a bilingual speakers [28]. However, the evidence to date is limited to participants who speak languages from the same linguistic family.

Our aim in the present report is to investigate the written and oral naming of actions and objects in a bilingual speaker with aphasia in two languages from different linguistic families: (Standard Modern) Greek and English. Unlike Spanish and Catalan, these languages differ in a number of features including morphological complexity, orthographic transparency and the type of script that is used for reading and writing. The key linguistic differences between Greek and English are summarized in Table 1. Unlike English, which is characterized as a word-based language, Greek is a stem-based language with a relatively complex morphology [13]. As in some English words, morpho-phonological word forms are inflected according to *grammatical category*, for instance '*skoup-izi*' is a verb [translation '*he/she sweeps*'] and '*skoup-a*' is a noun [translation '*broom*']. Nouns and verbs are differentiated by suffixes and marked for person and gender. Each stem in Greek is bound and presented for correct output after the correct inflectional suffix is attached to the stem at the morphological level. The Greek gender system with masculine, feminine and neuter gender is relatively complex. Nouns inflect gender at a morphological level. However, nouns are not only assigned gender on the basis of word meaning, but also on the basis of more general morphological rules. For instance, nouns ending with the nominative singular suffix –as ('*o papas*' [translation '*the priest*']) and with the accusative singular –a ('*ton papa*') are masculine, whereas nouns ending with the nominative singular –as ('*to kreas*' [translation '*the meat*']) and the accusative singular –as ('*to kreas*') are neuter. Gender agreement is also marked in Greek throughout the whole noun phrase, including determiners and adjectives. Information about the grammatical category and morpho-syntactic features of a Greek word, such as person, tense and mood for verbs or gender and case for nouns, are prominent aspects in the language as they must be accurately projected, marked and expressed during single word production. Thus, grammatical information can be assumed *prima facie* to be a constraint on selection and retrieval of appropriate phonological or orthographic representations (or lexeme) for output via language-specific processes necessary for morpho-phonological and morpho-orthographic encoding.

All cognitive models of lexical retrieval assume that spoken and written word production share conceptual representations but, post-semantically, to have different output mechanisms [2]. To retrieve a picture name, the speaker must first identify an action or object (e.g. a picture of scissors) and recognize the concept. This is considered a *pre-semantic* stage of processing. Next, a name is retrieved after the concept is distinguished the visually and semantically similar items. This is called the *lexical-semantic* stage. It is at this stage that morphosyntactic information first becomes available; e.g. in English and Greek, *scissors* is a single noun and in Greek it comes with neuter gender. Finally, the phonological or orthographic form of the word *scissors* is delivered. For spoken naming, the corresponding representation of the sound of the word is stored in the phonological output lexicon and instructions are given to the sensori-motor system to coordinate and produce speech sounds. For written naming, a corresponding orthographic form is retrieved from the orthographic output lexicon, which activates graphemic patterns for written output including allograph selection, retrieval of graphic motor patterns and motoric execution [5].

One theoretical issue in the study of lexical retrieval during written word production is the question of whether the sound of a word must be activated prior to retrieval of orthographic information. The Obligatory Phonological Mediation Hypothesis (OPMH) assumes the mapping of a lexical-semantic structure to an orthographic word form in the orthographic output lexicon first *requires* access to the phonological output lexicon. By contrast, the Orthographic Autonomy Hypothesis (OAH) assumes that orthographic representa-

Table 1
Key differences between the orthographic and phonological systems of Greek and English

| Language system | Greek | English |
|---|---|---|
| **Orthographic system** | | |
| Orthography | Transparent | Non-transparent |
| Letter inventory | More characters/letters than corresponding sounds | Fewer characters/letters than corresponding sounds |
| Orthographic principle | Phonemic basis | Largely morphophonemic basis |
| Alphabetic script | Mapping Greek letters to Greek phonemes | Mapping Roman letters to English phonemes |
| **Phonological system** | | |
| Vowel inventory size and complexity | Unmarked five-vowel system with vowels evenly distributed in the periphery of the vowel space. | Complex and highly marked vowel system with over 20 vowels (depending on dialect) comprising of both monophthongs that mark length and diphthongs. |
| Prosody | Trochaic but syllable-timed | Trochaic but stress-timed |

Table 2
Self – rated estimates of proficiency in his different languages across linguistic domains and of language use (percentage)

| | Estimates of proficiency | | | | | Language use background (percentage) | | | | |
|---|---|---|---|---|---|---|---|---|---|---|
| | Understanding | Speaking | Reading | Writing | | Home life | Tertiary education | Socializing | Reading | TV/ Radio |
| L1 (Greek) | 7.0 | 7.0 | 7.0 | 7.0 | L1 (Greek) | 75% | 100% | 75% | 75% | 12.5% |
| L2 (English) | 6.0 | 7.0 | 5.0 | 6.0 | L2 (English) | 12.5% | – | 12.5% | 25% | 75% |
| L3 (Arabic) | 5.0 | 5.0 | – | – | L3 (Arabic) | 12.5% | – | 12.5% | – | 12.5% |

Key: 5 = good abilities, 6 = very good abilities, 7 = excellent (native-like) abilities.

tions are directly retrieved without any phonological mediation [33]. These theoretical views make different predictions about the effect of grammatical class on written word output. According to the OPMH, grammatical class effects in written word production should be accompanied by an effect of grammatical class on spoken word production. However, the OAH makes no such prediction allowing for dissociable effects. Any dissociation between action and object naming in written and spoken word output is strong evidence against the OPMH. If such dissociations can be found in a bilingual speaker, this would be evidence that grammatical category is an organizing principle of the language system shared across languages as argued by Miozzo et al. [28, see also 17]. We report such evidence below.

## 2. Case report

AA is a 25-year-old right-handed male student who has a left parieto-occipital lesion verified by neuroimaging (CT) as a result of cerebral hemorrhage on 19 February 2006, while sleeping. An angiography conducted on 21 February 2006 revealed an arteriovenous malformation (AVM). AA has a right hemi paresis involving the arm and leg. He was right-handed before the stroke but now writes only with his left hand. AA was born in Athens, Greece, and raised as a simultaneous bilingual speaking Greek, English and Arabic from infancy. However, he was educated in Greek only with some years of English language instruction during schooling in Greece. AA's mother is Greek, his father Palestinian and the home language is English. He has two younger brothers. AA was asked to rate his abilities in each language prior to and post stroke and the results of these ratings are presented in Table 2. His family members verified the responses. AA was asked to quantify his current domain of language use. This information is presented in Table 2. AA considers Greek his predominant language and English his second language. Hence we classified Greek as L1 and English L2. AA had no premorbid writing disorder and vision and his hearing were normal. At the time of testing he was in the third year of a 4-year bachelor degree (Building Renovation and Restoration at the Technological Educational Institute Patras), and he read and wrote in Greek on a daily basis. This course contains three English language modules of graded difficulty, which AA completed prior to his stroke. A (bilingual) language questionnaire [26] administered in Greek was used to assess his language background. Information included the age of language acquisition, domains of language use and estimated proficiency in each language [30].

### 2.1. Neuropsychological evaluation

During collection of demographic information and checklist completion, AA was able to provide personal information such as age, nationality, education and family history of neurological and psychiatric conditions. He was co-operative with testing. AA's understanding of task instructions was good. Premorbid intellectual ability was estimated using subtests (Vocabulary and Matrix Reasoning) taken from the Wechsler Abbreviated Scale of Intelligence (WASI) [27,35] giving a Full Scale IQ score of 95 ($37^{th}$ percentile). His score on the Greek adapted version of the Mini Mental State Examination (MMSE) [6] was 29/30 (absence of dementia). AA's verbal and visual memory was assessed with the CNS Vital Signs memory test, which is translated into Greek [9]. In this test participants must learn 15 words and 15 geometric figures and recognize them from distractors. This task has immediate and delayed recognition/recall trials. AA obtained a standard score (age and education adjusted) of 110 on the verbal immediate recall trial ($75^{th}$ percentile rank for his age and education level) and 97 on the verbal delay recall trial ($42^{nd}$ percentile rank for his age and education level) indicating an average overall verbal memory performance, but difficulties in recalling previously learned information. On visual memory testing, his standard score (age and education adjusted) was 107 on the immediate recall trial ($68^{th}$ percentile rank for his age and education level) and 87 on the visual delay recall trial ($19^{th}$ percentile rank for his age and education level). This is in the low average range, especially recall of visual information after a delay. Executive functions were assessed with the CNS Vital Signs STROOP Test [9]. This is a measure of goal directed behaviour and flexibility required for self-correction. His score was in the impaired range between $1^{st}$ and $13^{th}$ percentile age and education adjusted. The CNS Vital Signs Symbol Digit Coding Test [9] was also administered to assess psychomotor speed and visual-motor coordination. Performance was poor ($1^{st}$ percentile age and education adjusted) showing slowed information processing when perceiving, attending and responding to visual information. We assessed his mood with the Beck Depression Inventory – Fast Screen (BDI-FS) [1] and the Beck Anxiety Inventory (BAI) [1]. AA scored 5/21 on the BDI-FS and 14/63 on the BAI, showing no significant depression but some mild anxiety.

### 2.2. Language assessment

Language testing was carried out in a quiet treatment room at the Speech and Language Clinic of the Technological Educational Institute (TEI) Patras. AA was tested over three-months. He was administered (a) the Boston Diagnostic Aphasia Examination (BDAE) in English [8] and Greek [29] to determine aphasia type and severity; (b) Greek Object and Action Test (GOAT) [21] of spoken and written retrieval of action and object names; and (c) phonological and orthographic processing subtests of the Athina Diagnostic Test of Learning Difficulties [31].[1] To test spelling, AA was given (i) nonwords in Greek and English to test sub-lexical/phonological spelling ability and (ii) homophones and cognate words to test lexical-semantic/non-phonological processing. A Greek-English speaker (the first author) administered all tasks. According to the BDAE, AA presented with anomic aphasia characterized by fluent, well-articulated speech but mild to moderate anomia (word-finding difficulties) [7] in Greek (L1) and English (L2). His auditory comprehension across language tasks was within normal limits in both languages. As writing impairments are our focus, writing subtests from the BDAE were administered. There was no evidence of motor writing impairments and we note that AA had trained himself to write with his non-paretic (left) hand. He showed a good writing style with well-formed letters and legible cursive writing. As a full-time university student, AA reported spending many hours per day writing (by hand) in Greek and also in English. In both scripts, on all tasks, AA wrote in lower case letters only. Writing was assessed on the following tasks in Greek and English: a) mechanics of writing (automatic writing: name and address) including recall of written symbols (serial writing-alphabet and numbers; primer-level dictation-writing individual numbers, letters, primer words to dictation; b) written word finding (spelling to dictation and written confrontation naming); and c) written formulation (narrative writing). These results are summarized in Table 3.

### 2.3. Tests of hypotheses

Previous studies of bilingual speakers have reported effects of grammatical class on spoken and written word

[1] The Athina Diagnostic Test of Learning Difficulties is partly based on the Illinois Test of Psycholinguistic Abilities and is standardized on healthy Greek speakers.

Table 3
AA's results on the writing subtests of the BDAE in L1 and L2

| Subtest | Greek (L1) | English (L2) |
|---|---|---|
| Automatic writing (name and address) | Judged to be similar to pre-morbid writing | Judged to be similar to pre-morbid writing |
| Recall of alphabet | 24/24 correct | 8/26 correct |
| Recall of numbers | 21/21 correct | 21/21 correct |
| Writing letters | No impairment | No impairment |
| Writing numbers | No impairment | No impairment |
| Copying | No impairment | 1 letter substitution (dog ⇒ bog) |
| Written word finding | 10/10 | 2/10 |
| Written picture description "Cookie Theft" | Organized coherent account: 31 words, 1 spelling error | Organized coherent account: 24 words, 7 spelling errors, 1 grammatical error |

naming with better performance for actions than objects in both languages [11]. According to the obligatory phonological mediation hypothesis (OPMH), effects of grammatical class on written word naming must be accompanied by the same effect on spoken word naming. Our first goal was to assess whether AA was impaired on lexical retrieval tasks. We did this with subtests from the Greek Object and Action Test (GOAT) [21] administered to assess spoken and written naming of actions and objects. The GOAT has 84 items: 42 action and 42 object colored photographs measuring 10 $\times$ 14 cm in size. No items were cognates in Greek and English [30]. Pictured actions were mono-transitive verbs. Actions included instrumental verbs (sweeping) and non-instrumental verbs (climbing). Objects were all inanimate nouns. There were no significant differences in mean word frequency, age of acquisition (AoA) or stimulus length between nouns and verbs in Greek (see Appendix 1) and no differences in written frequency in English between nouns (mean 89.31 per million) and verbs (mean 69.95 per million) on GOAT subtests [35]. Responses were recorded via audiotape. Error coding was based on a standard system [24].

### 2.4. Spoken and written naming

AA was shown photographs individually and asked to describe the picture using one word either in Greek or English, according to language tested. Each language was tested at least one week apart and all subtests presented in random order. Oral and written naming was tested separately.

AA was able to produce the correct spoken name in Greek for 19/42 (45.2%) actions and 17/42 (40.5%) objects and in English for 12/42 (28.5%) actions and 12/42 (28.5%) objects. Chi-square analysis found a significant difference in spoken naming between Greek and English $\chi^2 = 21.42$ $p < 0.001$, with better performance in Greek compared to English for actions $\chi^2 = 8.67$, $p = 0.003$ and objects $\chi^2 = 27.42$, $p < 0.001$. Within languages, there were no significant differences in action and object naming. In Greek, "Don't know" responses were dominant for actions 13/23 (56.5%) and objects 7/25 (28%) although semantic errors 8/25 (32%) and code-switching errors 5/25 (20%) were observed. In English, "Don't know" responses were dominant for actions 16/25 (64%) followed by semantic errors 7/23 (30.4%) and omissions 6/25 (24%) whereas for objects, omissions were dominant 24/29 (83%).

AA was able to produce the correct written name in Greek for 34/42 (81.0%) actions and 28/42 (66.6%) objects and in English for 8/42 (19.0%) actions and 8/42 (19.0)% objects. Chi-square analysis found a significant difference in written naming between Greek and English $\chi^2 = 3.42, p < 0.001$, with better performance in Greek compared to English for actions $\chi^2 = 5.25$, $p = 0.022$ and objects $\chi^2 = 42.85$, $p < 0.001$. There was a significant difference in written performance for actions compared to objects in Greek $\chi^2 = 5.95$, $p < 0.001$, with better performance for actions. There was no significant difference in action and object written naming in English. In Greek, the predominant error type was letter substitutions when writing actions (7/7) and objects (13/14). Greek errors involved substitution of a single vowel in either word initial (3/7) or word medial position (4/7) for action and word final (8/13), initial (3/13), and both initial and final positions in the same word (2/13), for object words. Moreover, all written naming errors for actions and objects were phonologically plausible alternatives in Greek. In English, errors with action and object names were mostly omissions and performance for both word types was close to the floor.

## 3. Discussion

We observed differential effects of word class on spoken and written word retrieval: an effect of grammatical class on written word production but not spoken word production in Greek and no effect of word class on spoken or written word retrieval in English. Our findings contrast with previous studies of bilingual speakers reporting effects of grammatical class on spoken word *and* written word naming with superior performance on nouns compared to actions in each language.

AA was better at writing action than object names in his native language (Greek) only. According to the obligatory phonological mediation hypothesis (OPMH), effects of grammatical class on written word naming must be accompanied by the same effect on spoken word naming. We therefore reject the OPMH as an explanation for written word naming in bilingual speakers. In addition to the effect of grammatical class on writing in Greek, AA was more impaired at spoken (43% correct) than written naming (73% correct) in Greek with no differences between spoken (28.5%) and written naming in English (19%). We assume there was damage at the level of the phonological output lexicon and the orthographic output lexicon in Greek and English (see Fig. 1) given AA has normal comprehension in both languages. Semantic errors in spoken naming were observed in both languages when the target was unavailable [2,19]. However, semantic errors were absent in writing. Writing errors were more likely to reflect knowledge of phonology to orthography correspondences in both Greek and English. We take this dissociation in error types as evidence of selective damage to phonological and orthographic output lexica.

The orthographic autonomy hypothesis [33] can explain the effect of selective damage to the orthographic output lexicon and the effect of grammatical class on written word output in Greek. According to this account, separate input and output components are available for writing verbs and nouns in the orthographic output lexicon [18] as in the phonological output lexicon [14]. AA's writing of action names – although not perfect – was significantly better than writing object names. The dominant writing error for objects was letter substitutions i.e. phonological-orthographic errors, involving the vowel "i" [ι] (iota) in word final position replaced by the vowel "i" [η] (ita); iota is the most common ending for singular neuter nouns, while ita is the most common ending for singular feminine nouns in Greek. Writing errors on (neuter) object words in Greek could be called morphological as AA assigned feminine gender to neuter nouns. However, we can reject this account for two reasons: first, he retrieved the gender of every noun correctly (100%) on the spoken naming task when asked to provide gender by the examiner, and second, he was able to write other neuter nouns ending with *iota*, e.g. spoon, sponge, fishing rod. Furthermore, all other errors for objects in Greek involved letter substitutions or phonological-orthographic replacements of similar-sounding vowels (e.g., αναπτ**ύ**ρας instead of αναπτ**ή**ρας "lighter"). As Greek has a transparent orthography, one explanation for performance on written picture naming of Greek neuter nouns is that spelling proceeded via a sublexical route (phoneme to grapheme conversion), delivering phonologically plausible alternatives lacking word-specific orthographic knowledge. There are alternative spellings of the vowel "i" and other vowels in Greek [32]. Word-specific knowledge is required to write words containing (vowel) orthographic ambiguity. Moreover, it is likely that access to morphological rules for neuter noun declensions was partly available to AA (after stroke), which would explain his mixed written picture naming performance mainly on (neuter) nouns.

Errors in action word written naming also show phonological-orthographic substitutions of word-medial or final vowels (e.g., κλειδ**ό**νει for κλειδ**ώ**νει "locking", τρ**ι**πάει for τρ**υ**πάει "drilling") that are also phonologically plausible alternatives. We contend that AA's action word writing (close to ceiling) involves an orthographic lexicon for spelling (orthographic ambiguous) action words and b) contribution from sublexical language-specific phonological to orthographic conversion procedures for action words. There was also evidence that AA relied on a sublexical route to spell object names in Greek. He produced written errors on vowel phonemes that are represented by many spellings in Greek. Considering that the orthography of Greek words is based not only on the phonological but also on the etymological information of a word, it would be expected that AA developed a visual orthographic representation that allows him to write all known words, but particularly words with phonemes that are ambiguous, i.e. can be spelled with more than one grapheme. Relying on a phonological transcription strategy will not automatically give a correct spelling for Greek words. It is not clear why damage to the orthographic output lexicon would have a greater effect on writing object names compared to action names but some explanations can be excluded. There were no significant differences in mean word frequencies, age of acquisition or stimulus length between objects and actions in Greek. It is important to note that regularity of spelling in action and object names was not controlled, but this is irrelevant to explaining writing performance according to the OAH. The OAH assumes that writing picture names does not require access to phonological representations, which are necessary to spell from dictation and thus could lead to differential effects on the spelling of regular and irregular words. According to the OAH, damage to the orthographic output mechanism for writing objects must be more impaired for AA due to greater loss of word specific orthographic knowledge or due to greater damage to morpho-syntactic properties for the retrieval of nouns from the lexical system.

There was evidence of impairment to the lexical retrieval of nouns in spoken production. However, AA's performance on spoken word naming of objects (40.5%) was worse than written word naming of the same objects (66.6%) a pattern that was magnified for spoken word naming of actions (42.5%) compared to written word naming of actions (81%). Therefore, it is not likely that impaired writing of object names simply reflects AA's impairment in lexical retrieval only. We contend that effects of grammatical class on object naming in Greek reflect a loss of specific orthographic knowledge about linguistic features of Greek nouns. We noted above a tendency for AA to assign feminine gender when writing neuter nouns although he could retrieve the gender of every noun correctly on the spoken naming task. This suggests that AA has a specific problem with retrieval of knowledge about noun gender at the orthographic level. Of interest, preserved knowledge of orthography allowed AA to produce better written word naming than spoken word naming of both actions and objects, although superior written word naming always has a benefit from preserved knowledge of spoken as well as orthographic information (and this is not true in spoken picture naming).

AA demonstrated deficits across spoken and written production in English (L2) with differences in performance reflecting premorbid familiarity with written English. The absence of any effects of grammatical class in L2 when compared to the effect on written word naming in L1 does not challenge the OAH provided the hypothesis is able to assume selective damage across both languages of a bilingual speaker (as well as between word class and modality of output). An advantage for nouns over verbs in L1 and L2 written picture naming was reported for a bilingual Spanish-Catalan speaker, JPG [12]. The effect of word class was reported for spoken picture naming in both languages. In general, JPG's performance was qualitatively very similar across languages and tasks, but the magnitude of the effect was larger in Catalan (L2).

Dissociations between actions and objects in written and spoken word naming support the claim that grammatical category is an organizing principle of the language system. However, we predicted equivalent impairments across both languages of a bilingual speaker following Miozzo et al. [28]. The absence of any effect of grammatical class in L2 is not necessarily a problem for the grammatical processing hypothesis in bilingual aphasia. Effect of word class on spoken and written picture naming in English may be difficult to detect given that performance was so near to floor. However, this does not explain why action naming in L1 is better than action naming in L2 or effects of language on performance across tasks. One possibility is that these effects reflect impairment to cognition such as attention, executive function and memory. AA's recall of visual information after a delay was poor and executive functions was impaired, with slow information processing given visual information. Slow processing and impairment to delayed visual memory may have more impact on writing verbs and nouns in English than in Greek since there are more irregular words in English requiring retrieval of word specific orthographic knowledge. This is likely to affect performance on picture naming tasks because phonological input is limited. We contend that it is important to consider cognitive impairments when explaining grammatical class effects on performance in any language.

To conclude, the results support the view that word class is a constraint on lexical retrieval. The results can be explained by the OAH because effects of grammatical class were restricted to lexical retrieval of word specific orthographic knowledge in Greek. Differences in morphological and orthographic features between languages explain effects of word class on writing in bilingual dysgraphia. For most writers, word patterns of English are picked up during the acquisition of reading and writing skills and most are learned through familiarity with the alphabetic symbols and their pronunciations or by memorizing of whole-word forms.

## Appendix

Picture characteristics of the GOAT based on psycholinguistic variables: lemma frequency, syllable length, age-of-acquisition (mean years;months), word imageability (0 = not imageable, 7 = highly imageable) and picture complexity (0 = high complexity, 7 = low complexity).

| Picture type | Lemma frequency | Syllable length | Age-of-acquisition | Imageability | Picture complexity |
|---|---|---|---|---|---|
| Objects | 40.91 | 2.88 (0.80) | 2.98 (0.76) | 6.49 (0.49) | 6.49 (0.28) |
| Actions | 40.11 | 2.95 (0.73) | 2.82 (0.58) | 6.42 (0.16) | 6.16 (0.67) |

Key: ( ) = standard deviations.
